# Supplementary material for: Discovery and characterization of cross-reactive intrahepatic antibodies in severe alcoholic hepatitis
Source: eLife. 2023 Dec 6;12:RP86678. doi: 10.7554/eLife.86678 (PMC10699809; doi:10.7554/eLife.86678)
Supplement: Figure 4—source data 1. [file elife-86678-fig4-data1.docx]

**Figure 4 - Source Data 1.** The numbers of unique autoantigens recognized by antibodies extracted from the diseased liver tissues (HuProt arrays).

|  | **IgG-binding** | **IgA-binding** | **IgM-binding** | **IgE-binding** | **Total** |
| --- | --- | --- | --- | --- | --- |
| **SAH** | 188 | 45 | 7 | 0 | 240 |
| **AC** | 0 | 4 | 0 | 0 | 4 |
| **HBV** | 1 | 88 | 25 | 5 | 119 |
| **HCV** | 32 | 4 | 0 | 3 | 39 |
| **PBC** | 7 | 2 | 81 | 0 | 90 |
| **NASH** | 0 | 4 | 0 | 0 | 4 |
| **AIH** | 0 | 0 | 1 | 0 | 1 |
