# Supplementary material for: Discovery and characterization of cross-reactive intrahepatic antibodies in severe alcoholic hepatitis
Source: eLife. 2023 Dec 6;12:RP86678. doi: 10.7554/eLife.86678 (PMC10699809; doi:10.7554/eLife.86678)
Supplement: Figure 4—source data 2. [file elife-86678-fig4-data2.docx]

**Figure 4 – Source Data 2.** The numbers of unique *E. coli* antigens recognized by antibodies extracted from the diseased liver tissues (*E. coli* proteome arrays).

|  | **IgG-binding** | **IgA-binding** | **IgM-binding** | **IgE-binding** | **Total** |
| --- | --- | --- | --- | --- | --- |
| **SAH** | 53 | 110 | 54 | 45 | 262 |
| **AC** | 83 | 54 | 1 | 1 | 139 |
| **HBV** |  |  | 1 | 6 | 7 |
| **HCV** |  |  |  | 9 | 9 |
| **PBC** |  |  | 69 |  | 69 |
| **NASH** |  |  |  |  |  |
| **AIH** |  |  |  |  |  |
