# Supplementary material for: Discovery and characterization of cross-reactive intrahepatic antibodies in severe alcoholic hepatitis
Source: eLife. 2023 Dec 6;12:RP86678. doi: 10.7554/eLife.86678 (PMC10699809; doi:10.7554/eLife.86678)
Supplement: Figure 6—source data 1. [file elife-86678-fig6-data1.docx]

**Figure 6 – Source Data 1.** Cellular components recognized by IgM and *E. coli* enriched IgM extracted from PBC liver tissues.

Appendix 1 - Table 4A. Cellular components recognized by Ig from PBC livers

| **Antigen enriched cellular component** | **Golgi membrane** | **Palmitoyltransferase complex** | **Extracellular exosome** | **Intrinsic component of Golgi membrane** |
| --- | --- | --- | --- | --- |
| **PBC-IgM** | P<0.01 | P<0.01 | P<0.05 | P<0.05 |

Appendix 1 - Table 4B. Cellular components recognized by E. coli enriched Ig from PBC livers

| **Antigen enriched**  **cellular component** | **Cytosol** | **Protein acetyltransferase complex** |
| --- | --- | --- |
| **PBC-IgM** | P<0.001 | P<0.05 |
