# Supplementary material for: Discovery and characterization of cross-reactive intrahepatic antibodies in severe alcoholic hepatitis
Source: eLife. 2023 Dec 6;12:RP86678. doi: 10.7554/eLife.86678 (PMC10699809; doi:10.7554/eLife.86678)
Supplement: Figure 6—source data 2. [file elife-86678-fig6-data2.docx]

**Figure 6 – Source Data 2.** Cellular components recognized by IgG or IgA antibodies extracted from the diseased liver tissues (Observed vs. Expected)

| **Antigen enriched cellular component** | **Cytosol** | **Cytoplasm** | **Nucleus** | **Mitochondrion** | **Focal adhesion** | **Extracellular exosome** | **Mitochondrial intermembrane space** | **Ruffle membrane** |
| --- | --- | --- | --- | --- | --- | --- | --- | --- |
| **SAH (IgG)** | P<0.0001 | P<0.0001 | P<0.0001 | P<0.05 | P<0.05 | P<0.05 | P<0.05 | P<0.05 |
| **SAH (IgA)** | P<0.05 | P<0.05 | ns | ns | ns | ns | ns | ns |
| **AC (IgG)** | ns | ns | ns | ns | ns | ns | ns | ns |
| **AC (IgA)** | ns | ns | ns | ns | ns | ns | ns | ns |
| **HBV**  **(IgG or IgA)** | ns | ns | ns | ns | ns | ns | ns | ns |
| **HCV**  **(IgG or IgA)** | ns | ns | ns | ns | ns | ns | ns | ns |
| **NASH**  **(IgG or IgA)** | ns | ns | ns | ns | ns | ns | ns | ns |
| **AIH**  **(IgG or IgA)** | ns | ns | ns | ns | ns | ns | ns | ns |
