# Supplementary material for: Discovery and characterization of cross-reactive intrahepatic antibodies in severe alcoholic hepatitis
Source: eLife. 2023 Dec 6;12:RP86678. doi: 10.7554/eLife.86678 (PMC10699809; doi:10.7554/eLife.86678)
Supplement: Supplementary file 1. [file elife-86678-supp1.docx]

**Human protein (autoantigen) sets recognized by both Ig and E. coli-captured Ig from SAH livers**

| IgA | IgG | IgM | IgE |
| --- | --- | --- | --- |
| ACER2  ACRC  ACSL6  ACSS1  ADA  ADH5  AGTR1  AK4  AK6  AKAP8L  ALKBH2  AMD1  ANAPC15  ANKHD1  ANKRD17  ANKRD28  ANP32A  AP1G1  AP3B1  APIP  APMAP  APOBEC2  APOH  AQP5  ARHGAP1  ARHGAP4  ARHGEF3  ARHGEF39  ARMC9  ARPC4  ASB17  ASPHD2  ASTL  ATAD2  ATCAY  ATG10  ATG13  ATP5B  ATP5E  ATP6V1C1  ATXN3  B3GALT6  B4GALT3  BABAM1  BACH1  BC071784_frag  BC096236_frag  BCS1L  BDH2  BEGAIN  Bhlhe22  BIN1  BOLA3  BORA  BRAT1  BRDT  BT006925  BZW2  C11orf1  C12orf10  C12orf60  C14orf166  C15orf41  C16orf58  C16orf62  C16orf70  C17orf80  C18orf23_frag  C1orf112  C1orf43  C1orf94  C1QBP  C1QL1  C1QL3  C1RL  C4orf19  C4orf22  C5orf24  C6orf182  C8orf59  CALR  CAMK1G  CAMK4  CAPN6  CARD9  CASD1  CASK  CASQ2  CASS4  CAT  CCDC106  CCDC14  CCDC40  CCDC9  CCDC97  CCL21  CCNA1  CCND3  CCNDBP1  CCR5  CD209  CD2BP2  CD44  CD52  CDC23  CDC42BPA  CDCA7L  CDCP1  CDCP2  CDH12  CDH13  CDK9  CDKN1A  CDKN2B  CDKN2D  CDO1  CDR2L  CEACAM21  CENPB  CENPC  CENPH  CENPM  CENPT  CENPU  CEP112  CFHR5  CFP  CHCHD4  CHMP4B  CHRNA1  CKB  CKM  CLEC2D  CLEC4E  CLEC7A  CLLU1OS  CLP1  CLPB  CLUAP1  CMPK1  CNNM1  CNOT10  CNST  COBLL1  COL26A1  COL4A3BP  COL5A2  COLEC10  COMP  COMT  COPS7B  COPS8  COQ7  COX4I1  CPSF4  CREB1  CREB3L3  CRIP2  CRLF2  CRYBB3  CSH1  CSNK2B  CT45A1  CT45A3  CTCFL  CTRL  CTTN  CUTA  CWC27_frag  CYP20A1  CYP4F11  DAAM1  DAPP1  DBN1  DBP  DCAF8  DCK  DCTN3  DEF6_frag  DEFB112  DEFB129  DEFB136  DFFA  DGKG  DGUOK  DHRS2  DHX32  DIABLO  DMGDH  DMP1  DMRT2  DNAJA3  DNAJA4  DNAJC30  DPY19L3  DRAP1  DRC7  DRICH1  DTNBP1  DUSP22_frag  DUSP6  ECHDC3  ECHS1  ECI1  ECSIT  EDEM2  EEF1D  EEF1DP3  EEF2K  EGFR  EIF2B5  EIF3L  ELAVL3  ELMO1  ELP5  EMD  ENOPH1  EPHA8  EPO  EPS8L2  ERBB3  ERICH2  ERP27  ESM1  ESRRG  EXOSC7  EXTL3  F2  F8A1  FAM104B  FAM114A1  FAM120B  FAM131B  FAM131C  FAM13A  FAM156A  FAM21A  FAM29A  FAM45A  FAM9B  FAM9C  FAS  FAU  FBXO2  FBXO21  FBXO38  FCHO1  FEM1B  FEZF2  FGF12  FGF20  FGFBP3  FIGNL2  FN3K  FOLR2  FPR3  FSBP  FUCA2  FXYD5  G6PD  GAB1  GABPB1  GAR1  GATSL2  GCDH  GCK  GDAP1L1  GDF11  GGH  GHRH  GIMAP5  GIT2  GLB1  GLI1  GNA11  GOLPH3  GORASP1  GPATCH1  GPM6B  GPN1  GPT  GRK7  GSTA2  GSTM3  GSTM4  GTF2A1L  GTF2I  GTPBP8  GTSF1  GUCY2F  GYS1  H1F0  HACE1  HCLS1  HEATR3  HELT  HEMK1  HEXA  HIATL1  HINFP  HIST1H1A  HLA-F  HMGB2  HNRNPC  HOXB6  HPCAL4  HRAS  HRC  HSCB  HSF2  HSP90B1  HSPA6  HSPB1  HSPB6  HTN3  HVCN1  IARS  IBSP  IDH1  IDH3G  IDI1  IDUA  IFFO1  IFIT3  IGL  IKZF2  IL32  IL4I1  IL5RA  ILF2  ILVBL  IMPDH1  ING3  INPP5A  INPP5K  IP6K1  IQCF1  IQUB  IRX2  IRX5  IST1  ITGB1BP2  IZUMO4  JHU18929  JMJD6  KANSL3  KCNAB1  KCNAB2  KCNK17  KCNRG  KCTD17  KDM4D  KHDC1  KIAA0040  KIAA0513  KIAA1429  KJ898030  KJ901255_frag  KJ901395  KJ901766.1_frag  KLC4  KLF7  KLHDC4  KLHDC7B  KLHDC8A  KRAS  KRT37  KRTAP10-6  KRTAP16-1  KRTAP3-2  LAP3  LARP1_frag  LARP4  LARS2  LAX1  LCE2C  LDOC1  LETM2  LIMK1  LIMK2  LINC00305  LINC01465  LOR  LPL  LRPAP1  LSG1  LTB4R  LUC7L  LY6D  LYN  LYSMD2  MAG  MAGEA6  MAGEA8  MAGEA9  MAGEC2  MAN2B1  MAP3K7  MAPK8IP2  MAPRE1  MASP1  MATK  MBTPS2  MED22  MED4  MEIOC  METTL14  METTL16  MGAT4A  MINK1  MLF2  MLST8  MMP28  MMRN2  MMTAG2  MRAS  MRPS27  MS4A12  MTBP  MTERF4  MTSS1L  MTX3  MYLK  MYRIP  MYT1  N4BP1  NAGK  NAMPT  NANS  NAPG  NCAPH  NCL  NCLN  NCS1  NDN  NDRG4  NDUFB11  NDUFB2  NDUFB5  NDUFS1  NEK11  NFE2  NFKB2  NFKBIB  NINJ1  NLN  NLRP13  NLRP14  NLRP5  NME2  NME7  NMNAT3  Nol3  NOL3  NOS1AP  NOSTRIN  NPM1  NPPA  NPY  NR2F1  Nr2f6  NSG1  NSMCE2  NTN4  NUMB  NUP107  NUTM2G  NXN  NXPH2  OBFC1  OCLN  OLIG3  Onecut1  OR10T2  OR5H6  OR9G1  ORC4L  OSBPL8  OTUD6B  OTX2  P3H4  PACSIN1  PAF1  PAFAH1B3  PAIP1  PAK3  PAK4  PAK7  PANK3  PARP8  PATZ1  PBDC1  PBX1  PBX4  PCDH17  PCDHB15  PCMTD1  PDE4DIP  PDE8A_frag  PDIA2  PDK1  PDS5B  PDXDC1  PDZK1IP1  PENK  PEX10  PFKFB4  PGA4  PGK1  PGRMC1  PHYHD1  PI4K2A  PICK1  PIM2  PIP4K2C  PLA2G2E  PLD3  PLEKHF2  PLEKHJ1  PLOD2  PNKD  PNMA2  PNMA6A  POGZ  POLR2F  POLR2J2  POTEE  PPBP  PPIC  PPM1G  PPP4R3A  PPP6R2  PPRC1  PRELID3B  PREX2  PRKAR2B  PRKCDBP  PRKD2  PRMT2  PRMT3  PRMT8  PRPH2  PRPSAP2  PRR13  PRRC2B  PSMA3  PSMC3  PSMD5  PTGES2  PTGIS  PTMA  PTMS  PTPN12  PTPN6  PTRH2  PTTG1  PUDP  PURA  PURG  Q6ir13_frag  RAB14  RAB21  RAB33A  RAD23A  RAD51D  RALY  RANGAP1  RAP1GDS1  RARA  RASGRP3  RASL11B  RASSF3  RBAKDN  RBM34  RBPMS2  RCHY1  RDM1  RFX8  RHOA  RHOQ  RILPL2  RIOK3  RIPPLY1  RLN3  RNF157  RNF25  RNFT1  ROBO3  RPL13  RPL36AL  RPLP1  RPS15A  RPSA  RRAGD  RRAS2  RRP15  RSPO4  RSRP1  RTN2  RTN4IP1  RUVBL2  S100A7L2  SAA2  SAMD4A_frag  SARAF  SARS  SCML1  SENP7  SESTD1  SET  SFR1  SGCD  SGK2  SGK494  SH2D3A  SH3BGR  SH3GL2  SHANK2  SHCBP1  SHFM1  SIRT4  SLAMF6  SLC16A8  SLC17A4  SLC1A7  SLC35E3  SLC37A2  SLC41A3  SLC48A1  SLC6A6  SLC7A5  SLC7A6OS  SLX1A  SMAD7  SMAD9  SMARCC2  SMC5  SMDT1  SMOX  SMYD5_frag  SNAI3  SNRNP27  SNRPF  SORBS2  SPACA7  SPAG11A  SPANXN2  SPANXN3  SPCS2  SPNS3  SPP1  SRGN  SRMS  SRPK2  SRSF11  SSC5D_frag  ST3GAL3  STARD3NL  STARD7  STAT4  STBD1  STK3  STK31  STK39  SUFU  SUGCT  SULT2B1  SUMO1  Supt6h  SUV39H2  SUV420H1_frag  SYT10  TAB2  TARBP1  TARBP2  TAZ  TBC1D9B  TBRG1  TBX19  TBX4  TCIRG1  TCN2  TCP1  TEK  TEN1  TERF2IP  TESK1  TEX101  TEX33  TFIP11  TGIF2LY  TGM1  TGM2  THY1  TIGD1  TIMM44  TIRAP  TLDC1  TLE4  TLK1  TLR4  TMEM101  TMEM106B  TMEM129  TMEM154  TMEM161B  TMEM168_frag  TMEM222  TMEM44  TMSB4Y  TNFRSF14  TNFRSF25  TNK1  TNNT2  TOM1L2  TONSL  TP73  TPD52L1  TPK1  TPTE  TRAPPC10  TRAPPC12  TSPAN18  TSPAN7  TSPY3  TSR2  TTC27  TUBA3C  TUBA8  TUBB  TULP3  TYROBP  TYW3  U2AF1  U2AF2  U2SURP  UBA5  UBALD2  UBE2D3  UBE2R2  UBE3A  UBXN4  UFD1L  UNC45A  USO1  USP14  USP25  USP4  USP7  VAMP3  VCP  VEGFB  VMA21  VPS16  VWA3B  VWA8  VWA9  WBP11  WDR55  WDR70_frag  WISP2  XAGE1A  XM_006509802.2_frag  XPA  XRCC4  YARS  YKT6  YWHAB  YWHAQ  YWHAZ  ZADH2  ZBED4  ZBTB7C  ZDHHC5  ZDHHC9  ZFPM2  ZKSCAN3  ZMAT4  ZMYM3  ZNF192P1_frag  ZNF207  ZNF225  ZNF326  ZNF330  ZNF346  ZNF415  ZNF428  ZNF460  ZNF557  ZNF843  ZSWIM1 | AAMP  AAR2  AARSD1  ABI1  ABR  ABTB1  ACER2  ACRC  ACSL6  ADH5  AGTR1  AHCY  AHNAK2  AK6  AKAP8L  ALPP  AMBN  ANAPC15  ANKHD1  ANKRD17  ANP32A  ANXA10  AOC2  AP1G1  AP3B1  APMAP  APOBEC2  APOH  APPL2  ARHGAP1  ARHGAP4  ARHGEF3  ARHGEF4  ARMC9  ARPC4  ASCL4  ASRGL1  ASTL  ATCAY  ATF2  ATG10  ATP12A  ATP8B5P_frag  ATXN3  B3GALT6  BAFF  BC031259.1_frag  BC034142.1_frag  BC054893.1_frag  BC070352.1_frag  BC071784_frag  BC073937.1_frag  BC089413  BC089418  BC096236_frag  BCS1L  BEGAIN  Bhlhe22  BIN1  BOLL  BORA  BRAT1  BRDT  BZW2  C10orf62  C12orf10  C12orf60  C14orf166  C17orf80  C1orf112  C1orf94  C1QBP  C1QL1  C1QL3  C1QTNF2  C1RL  C4orf19  C4orf22  C6orf182  C8orf59  CACNG6  CAMK1G  CAMK4  CASK  CASQ1  CASQ2  CASS4  CCDC106  CCDC137  CCDC178  CCDC28A  CCDC9  CCDC97  CCNA1  CCNDBP1  CCR5  CD209  CD44  CDC27  CDC42BPA  CDCA7L  CDCP2  CDH12  CDH13  CDKN2B  CENPB  CENPH  CFHR5  CHRNA1  CKM  CLEC7A  CLLU1OS  CLP1  CLPB  CLUAP1  CMC4  CNNM1  CNOT10  CNST  COL26A1  COL4A3BP  COMP  COMT  COPS8  COQ7  COX18  CPSF3  CPSF4  CREB3L3  CREBZF  CRLF2  CRNN  CSNK2B  CTCFL  CTTN  CUTA  CYB561A3  CYP2E1  CYP4F11  DAAM1  DAPP1  DBN1  DBP  DCAF8  DCK  DEFB112  DEFB129  DEFB131  DEFB132  DENND5A  DGKG  DHRS2  DHX32  DIXDC1  DMGDH  DMP1  DMRT2  DMRT3  DMRTB1  DMTF1  DNAJA4  DNAJB5  DNAJC11  DNAJC6  DPP3  DRAP1  DRC7  DRICH1  DTNBP1  DUB3  DUSP22_frag  E2f5  ECE1  ECHDC3  ECI1  ECSIT  EEF1DP3  EEF2K  EFCAB13  EGFR  EIF2AK4  EIF2B5  EIF3L  ELMO1  ELP5  EMD  ENOPH1  EPHA8  EPO  ERCC8  ERICH2  ERP27  ESM1  ESRRG  EXOSC7  F8A1  FAF1  FAM117B_frag  FAM120B  FAM124B  FAM129A  FAM131B  FAM131C  FAM13A  FAM21A  FAM29A  FAM45A  FAM9B  FAM9C  FAS  FBXO2  FCGR2A  FCHO1  FCHSD2  FEM1B  FEZF2  FGF12  FGF20  FIGNL2  FOLR2  FOXRED2  FSBP  G6PD  GAB1  GAR1  GARS  GATSL2  GCDH  GCK  GDAP1L1  GDPD5  GGH  GHRH  GLB1  GLI1  GNA11  GORASP1  GP1BB  GPM6B  GPN1  GPT  GRK7  GSN  GSTA2  GSTM4  GTF2I  GTSF1  GUCY2F  HACE1  HAX1  HCLS1  HCRT  HEATR3  HERC3  HIATL1  HINFP  HIST1H1A  HIST1H2BC  HNRNPC  HPCAL4  HRAS  HRC  HSCB  HSF2  HSP90B1  HSPA1A  HSPA6  HTN3  HVCN1  IARS  IBSP  ICAM4  IDH1  IDH3G  IDI1  IDUA  IFFO1  IFIT5  IGK  IGL  IL32  INPP5A  INPP5K  IP6K1  IQUB  IRS4  IRX2  IRX5  ISCU  IST1  ITGB1BP2  IZUMO4  JHU18929  JMJD6  JMJD7  KANSL3  KCNAB1  KCNK17  KCTD17  KDM4D  KIAA0040  KIAA0408  KIAA0513  KIAA1429  KIF19  KJ898030  KJ900918_frag  KJ901255_frag  KJ901395  KJ901523  KJ901766.1_frag  KLC4  KLF7  KLHDC4  KLHDC7B  KLHDC8A  KRAS  KRBA2  KRT37  KRT85  KRTAP10-6  KRTAP3-2  KRTAP6-3  KV205  LAGE3  LARP4  LAX1  LCE1E  LCE2C  LDOC1  LETM2  LIMK1  LIPM  LPAR4  LPL  LRPAP1  LSG1  LTB4R  LUC7L  LYN  LYSMD2  MAFG  MAGEA1  MAGEA6  MAGEA8  MAGEA9  MAGEC2  MAGI1  MAK16  MAN2B1  MAP2K2  MAP3K10  MAP4K1  MAPK8IP2  MAPRE1  MARC2  MASP1  MATK  MBIP  MBTPS2  MED4  MEIS3  METTL14  METTL15  METTL16  MFF  MIB2  MINK1  MMD2  MMRN2  MRAS  MRI1  MRPL1  MS4A12  MTBP  MTERF4  MTMR14  MTX3  MYLIP  MYRIP  MYT1  N4BP1  NAGK  NAMPT  NAPG  NCL  NCLN  NDN  NDUFB11  NDUFB2  NEDD4L  NFKB2  NFKBIB  NKAPL  NLN  NLRP13  NLRP14  NLRP5  NME2  Nol3  NOL3  NOS1AP  NOSTRIN  NOX1  NPM1  NPY  Nr2f6  NRCAM  NSG1  NSMCE2  NTN4  NUDT11  NUP107  NUTM2G  NXN  NXPH2  OBFC1  OCEL1  OCLN  OGFOD3  OGG1  OLIG3  Onecut1  OR10T2  OR9G1  ORC4L  OSTCP1  OTUD6B  P4HA3  PACSIN1  PAF1  PAH  PAIP1  PAK3  PAK4  PAK7  PALD1  PANK3  PARD3B  PARP8  PARS2  PBDC1  PBX1  PBX4  PCDH17  PCDHA4  PCDHB15  PCMTD1  PCSK7  PDE1A  PDE8A_frag  PDIA2  PDK1  PDK2  PDPK1  PDXDC1  PENK  PEX10  PFKFB4  PGA3  PGA4  PGK1  PI4K2A  PICK1  PIK3R5  PIP4K2C  PKNOX1  PLAA  PMVK  PNKD  PNMA2  PNMA6A  POGZ  POTEE  PPIC  PPM1G  PPP2R2B  PPP2R3C  PPP2R4_frag  PPP6R2  PRDM15  PREX2  PRH1  PRKAR2B  PRKCDBP  PRKCZ  PRMT2  PRMT3  PRMT8  PRPH2  PRPSAP2  PRR13  PRR16  PSMA3  PSMB6  PSMC3  PSMD5  PTGES2  PTGIS  PTMA  PTMS  PTPN6  PTRH2  PURA  RAB14  RAB21  RAB28  RAD23A  RALY  RANGAP1  RARA  RASGRP3  RBAKDN  RBM39  RCHY1  RDM1  RFX8  RHOA  RHOQ  RHOXF2  RIC8A  RLN3  RNF25  RNFT1  RNPEP  ROBO3  RPL36AL  RPLP1  RSPO4  RTN4IP1  RUVBL2  SAA2  SASH3  SCLT1  SENP7  SESN2  SESTD1  SET  SGK2  SH2D3A  SH3BGR  SH3GL1  SH3GL2  SHCBP1  SHFM1  SIRPB1  SIRT4  SLAMF6  SLC16A8  SLC16A9  SLC35E3  SLC6A6  SLC6A7  SLC7A10  SLC7A6OS  SMAD7  SMC5  SMOX  SMYD5_frag  SNAI3  SNX33  SOGA3  SPAG11A  SPANXN2  SPANXN3  SPNS3  SPP1  SRGN  SRMS  SRPK2  SRSF11  SRSF2  SSBP1  SST  ST3GAL3  STARD3NL  STARD7  STAT4  STK31  STK38L  STK39  STOX1  STRA13  SUCLG2  SUFU  SUMO1  Supt6h  TAB2  TAPBP  TARBP1  TARBP2  TAZ  TBC1D9B  TBX19  TBX4  TCF7L1  TCN2  TCP1  TEK  TEN1  TERF1  TERF2IP  TESK1  TEX33  TFIP11  TGIF2LY  TGM1  TGM2  THEMIS2_frag  TIGD1  TIRAP  TLE4  TLK1  TMEM106B  TMEM116  TMEM129  TMEM130  TMEM161B  TMEM209  TMEM222  TMEM44  TNFRSF14  TNFRSF25  TNK1  TOM1L2  TONSL  TOP1MT  TPK1  TPM2  TPSAB1  TRAPPC10  TRAPPC12  TRGC1  TRIM65  TSPAN10  TSR2  TTC27  TUBA3C  TUBA8  TUBB  TUBB8  TULP3  TYW3  U2AF1  U2SURP  UBALD2  UBE2D3  UBE2R2  UBE3A  UBXN4  UFD1L  UGT2B15  UNC45A  USP32  USP4  USP7  VCP  VDAC1  VRK2  VWA8  VWA9  WBP11  WDR55  WISP2  XM_006509802.2_frag  XPOT  XRN2  YARS  YIF1B  YKT6  YWHAQ  YWHAZ  ZADH2  ZBED4  ZBTB47  ZBTB7C  ZDHHC5  ZDHHC9  ZFPM2  ZMAT4  ZNF192P1_frag  ZNF207  ZNF225  ZNF254  ZNF326  ZNF330  ZNF428  ZNF460  ZNF598  ZNF687  ZNF746  ZNF772  ZNF843  ZSWIM1 | A0jns7  AAK1  ABI1  ABTB1  ACCS  ACER2  ACOT7  ACRC  ACSBG1  AFM  AFMID  AGTR1  AK094777.1_frag  AK2  AK4  ANAPC15  ANKHD1  ANKRD1  ANP32A  AP1B1  APIP  APOBEC2  APOC3  APOC4  APOH  APOL2  ARAF  ARF6  ARL17A_frag  ARL4D  ARL8B  ARMC9  ASB17  ATCAY  ATG13  ATIC  ATRIP  BABAM1  BACH1  BC002963  BC073758  BC073767  BC089412.1_frag  BCAR3  BCAS2  BCS1L  BEGAIN  BID  BMP7  BMX  BRAT1  BZW2  C11orf1  C12orf60  C16orf62  C17orf62  C1orf112  C1orf43  C1orf94  C1QBP  C2orf27A  C4orf19  C5orf24  C8orf59  C9orf16  CAMK4  CAMKK2  CAPN6  CAPS  Carm1  CASS4  CCDC127  CCDC9  CCDC97  CCND2  CD44  CD52  CDC23  CDCP1  CDK9  CDKN1A  CDKN2B  CDKN2D  CEACAM21  CENPU  CHCHD4  CHKB  CHMP1A  CKB  CKM  CKS2  CLDN1  CLEC4E  CLUAP1  CMC4  CMPK1  CNNM1  CNST  COL4A3BP  COX18  CPA2  CPOX  CPTP_frag  CRELD1  CSNK1E  CSNK2B  CUTA  CX3CR1  CYB5D2  CYP4F11  DAAM1  DAB1  DAZ2  DBN1  DCAF8  DCTN3  DDC  DEFB112  DERL2  DMP1  DMRT2  DPY30  DRICH1  DTD1  DTNBP1  DUSP6  EAF1  ECHDC1  EEF1G  EEF2  EEF2K  EGFR  EIF2B5  EIF3L  ENOPH1  EPHA8  ERBB3  ERICH2  ERP27  ESM1  ETHE1  EXTL3  F2  FAM129A  FAM131B  FAM131C  FAM134C  FAM21A  FAM45A  FAM84A  FAS  FAU  FBXO2  FGF12  FGF20  FGFBP1  FGGY  FIGNL2  FKBP7  FOLR2  FRMD8  FTL  GALK2  GAP43  GCHFR  GCK  GIMAP2  GIT2  GLUL  GNPDA1  GRPEL2  GSK3B  GTF2I  GTPBP3  GTPBP8  GTSF1  GUK1  HAX1  HCLS1  HEATR3  HIATL1  HK2  HKDC1_frag  HMBS  HPR  HRAS  HRC  HSCB  HTRA4  IDI1  IFFO1  IFI35  IFIT3  IFT22  IGK  IGL  IKBKB  IL32  IP6K1  IRF2BP1  ITGB1BP2  ITIH5  JMJD6  KCNAB1  KCNAB2  KCNIP3  KEAP1  KHK  KIAA0513  KJ898030  KJ901215  KJ901255_frag  KJ901517  KJ901885  KJ902696  KJ904347_frag  KJ905802  KJ905803  KJ905804  KJ905805  KLF10  KLF6  KRAS  LARP4  LDHD  LDOC1  LINC01126_frag  LOR  LPAR2  LRRC43  LYG1  MAGEA11  MAGEA8  MAGEA9  MAGEC2  MAGI1  MAP3K6  MAP4  MAPK8IP2  MAPKAPK3  MATK  MED4  MED7  MEMO1  MKNK1  MPST  MTERF4  MTHFD1  MTURN  MX2  MYCL  MYL4  MYLK  N4BP1  NAGK  NCS1  NDFIP1  NDN  NDRG1_frag  NEK11  NFE2L2  NFKBIB  NID2  NIF3L1  NKIRAS2  NME2  NME3  Nol3  NOL3  NOSTRIN  NR1H3  NUDT14  NXPH2  OR2T35  OR4K5  OR5D14  OR9G1  PACSIN1  PAF1  PANK3  PATZ1  PCK2  PDIA2  PFKM  PGRMC1  PHB2  PHKG2  PICK1  PIGC  PIP4K2C  PKNOX1  PLCD4  PMS2P5  PNPO  POLL  POLR2J2  PPM1G  PPP1R3C  PRDX2  PRLHR  PRMT2  PRMT3  PRMT8  PROSC  PRPSAP1  PRRC2B  PRSS42  PRUNE2  PSMA3  PSMB4  PSMC3  PSMD3  PSMD5  PTMA  PTMS  PTPRE  QDPR  R3HDM2_frag  RAB14  RAB33A  RAB35  RAB3D  RAB8A  RABL2B  RACK1  RAD23A  RALY  RANGAP1  RAP1GDS1  RASD2  RASGRP3  RBKS  RBM34  RBPMS2  RHOA  RIPK2  RNF24  RPLP1  RPP40  RPS15A  RRAGA  RRAGD  RRAS2  RRP15  RSPO4  RTN4  RUVBL2  S100A10  S1PR5  SAMD4A_frag  SAMD4B  SAT1  SCD  SCGB1C2  SCLT1  SCX  SDCBP  SDHAF2  SESN2  SESTD1  SET  SFN  SFXN1  SGK1  SH3BGR  SHH  SIGIRR  SKAP2  SLC16A8  SLC1A5  SLC41A3  SLC6A6  SLC7A5  SLC7A6OS  SMUG1  SMYD5_frag  SNRNP27  SNX33  SPANXN2  SPANXN3  SPNS3  SPP1  SQSTM1  SRGN  SRMS  SSBP1  SSBP4  SSC5D_frag  STAC3  STARD3NL  STK3  SUDS3_frag  SUFU  Supt6h  SYF2  SYT17  TAGLN  TAOK3  TBC1D9B  TCP10L  TCP11  TEX33  TFG  TGM1  THOC1  TK1  TLE4  TMEM39B_frag  TMEM40  TMEM44  TMEM51  TNK2  TPM2  TPTE  TRAPPC12  TRIM39  TRIM51  TRIM74  TSC22D3  TSPAN18  TSPAN9  TUBA1C  TUBA8  TXNDC15  UBE2D4  UBE2I  UBE2R2  UBE3A  UHMK1  USP25  USP4  USP7  VAMP3  VDAC1  VWA7  VWA8  WBP11  WBSCR27  WDR55  XRCC4  YWHAB  ZADH2  ZC4H2  ZCCHC12  ZDHHC5  ZFP64  ZMYM3  ZMYM6  ZNF207  ZNF428  ZNF557 | BCS1L  C1orf94  CCR7  CELF6  COQ7  CRYZ  DAZ2  DDAH1  DECR2  DNAJB2  EGFR  ELAVL4  F2  FAM109B  FAM49B  FTL  HERPUD1  HMBS  KCNAB1  KCNAB2  KRTAP4-4  LOR  MECR  MTHFD1  NME2  NME4  PLA2G6  PRLHR  PRRC2B  QDPR  QKI  RABL2B  RBM38  RBMY1A1  RUNDC3B  SOHLH2  SSBP1  TAF9B  VWA8  YBX3  ZADH2  ZNF358 |
